# Developing a national undergraduate medical education pain management and substance use disorder curriculum to address the opioid crisis: a program evaluation pilot study

**DOI:** 10.1186/s12909-024-05181-z

**Published:** 2024-03-08

**Authors:** N. Dalgarno, J. Turnnidge, N. Cofie, R. van Wylick, J. Mulder, F. Kirby, A. Hastings-Truelove, L. Graves

**Affiliations:** 1 Office of Professional Development and Educational Scholarship, Queen’s University, 385 Princess Street, Kingston, ON K7L 1B9 Canada; 2 Department of Pediatrics, Queen’s University, 76 Stuart St, Kingston, ON K7L 2V7 Canada; 3 Association of Faculties of Medicine of Canada, 150 Rue Elgin Street, Ottawa, ON K2P 1L4 Canada; 4 Master of Health Professions Education, Queen’s University, 99 University Avenue, Kingston, ON K7L 3N6 Canada; 5 Family and Community Medicine, Western Michigan University Homer Stryker M.D. School of Medicine, 1000 Oakland Drive, Kalamazoo, MI 49008-8017 USA

**Keywords:** Opioids, Pain Management, Program Evaluation, Undergraduate Medical Education, Substance Use Disorder

## Abstract

**Background:**

Pain and addiction are one of the most common reasons for adults to seek health care, yet educational programs focused on pain are often underrepresented in medical school curricula. In January 2021, the Association of Faculties of Medicine of Canada (AFMC) launched an online national, bilingual, competency-based curriculum for undergraduate medical (UGME) students in pain management and substance use in response to the opioid crisis and to bridge the content gaps in programs across Canada. The purpose of this study is to evaluate the pilot of this national curriculum.

**Methods:**

UGME students, from across Canada, participated in the program evaluation by completing online pre- and post-program surveys that assessed the influence of the curriculum on participants’ knowledge as well as the value, usability, and feasibility of this curriculum.

**Results:**

Participants’ perceived confidence in their new knowledge and in utilizing resources required to maintain their knowledge significantly increased (75% and 51% respectively). Their perceived knowledge that addressed the 72 learning objectives within the curriculum significantly increased from pre- to post-program. Over 90% of participants reported that the curriculum was valuable, feasible, and usable. The most frequently discussed program strengths were the clear and comprehensive content, interactive and well-organized design, and relevance of curriculum content for future clinical practice. The overall weakness of the curriculum included the length, repetition of content, the lack of clarity and relevance of the assessment questions, end-user technology issues, and French translation discrepancies. Participant’s recommendations for improving the curriculum included streamlining content, addressing technology issues, and enhancing the clarity and relevance of assessment questions embedded within each of the modules.

**Conclusion:**

Participants agreed that an online pain management and substance use curriculum is a valuable, usable, and feasible learning opportunity. Given the severity of the opioid crisis in Canada, these online modules provide a curriculum that can be integrated into existing UGME programs or can provide self-directed learning.

**Supplementary Information:**

The online version contains supplementary material available at 10.1186/s12909-024-05181-z.

## Background

Canada is in the midst of an ongoing opioid crisis. In the 1990s, North American pharmaceutical companies developed and aggressively promoted multiple opioid formulations as “low risk, non-addictive, effective treatments for moderate pain” [1]. While criminal charges of misrepresenting and misbranding were laid against at least one of these formulations (OxyContin) in 2007 [2], the damage to the public health system had already been done. From January 2016 to June 2018, more than 9,000 Canadians died from apparent opioid-related harms [3]. Canada and the United States now have the highest rates of prescription opioid use in the world [4, 5].

Pain is one of the most common reasons for Canadians to seek health care, with one out of every five adults in Canada experiencing chronic pain [6]. Nonetheless, educational programs focused on pain and addiction management are often underrepresented in medical school curricula. For example, Ung et al. (2016) found that the majority of medical students failed to meet an acceptable level (80% or higher) on the Knowledge and Attitudes Survey Regarding Pain [7]. While approximately 80% of U.S. medical schools, and 92% of Canadian medical schools require students to attend at least one session on pain, the median hours of instruction on pain topics varied considerably between schools, and many topics included in the International Association for the Study of Pain core curriculum received little or no coverage [8]. In medical education, there is often a lack of specific curricula related to pain and addiction management. Rather, this content is taught across various disciplines including anaesthetics, palliative care, pharmacology, and during clinical skills sessions. This fragmented approach to delivering pain management content may lead to gaps in student knowledge [7]. Educational pain and addiction programs, such as the Arizona Pain and Addiction Curriculum [9], the Brown University’s Internal Medicine Addiction Fellowship program [10] and the Pennsylvania State curriculum on opioids and addiction [11] may play a key role in the comprehensive response to the opioid crisis by addressing training gaps and empowering future physicians with the necessary knowledge, skills, and resources to diagnose, treat, and manage pain and substance/opioid use disorder. To contribute to this response, the Association of Faculties of Medicine of Canada (AFMC) collaborated with pain management and substance use experts from all 17 Canadian medical schools to develop a competency-based, bilingual (French and English) curriculum for undergraduate medical education (UGME) to be disseminated, adapted, and implemented at all Canadian medical schools.

In 2017-18, the AFMC began the process of developing an UGME pain management and substance use disorder curriculum. Through a combination of an environmental scan, surveys, and stakeholder meetings, six competency areas were identified for the curriculum. These included: (a) the public health perspective, (b) core concepts in pain and management of pain, (c) pathophysiology of pain and pharmacology of opioids, (d) opioid prescribing, opioid stewardship in palliative care, and safe storage and disposal of opioids, (e) recognizing and managing opioid use disorder, and (f) cultural and legal considerations for enhancing competence. The curriculum, entitled *Pain Management and Substance Use Disorder* consists of 10 online modules across the six topic areas [12, 13] to be voluntarily integrated into the UGME curriculum at each of the 17 medical schools in Canada. The purpose of this paper is to report the evaluation findings of the newly developed curriculum which was evaluated from September 15 to November 15, 2020 (as part of pilot testing). The purpose of the program evaluation was to:

1. evaluate the extent to which this program is achieving its intended outcomes (enhance competencies, increase awareness, increase knowledge and skills in diagnosis, treatment, and management of patients with pain and substance use disorder).
2. evaluate the extent to which the program is meeting the needs of the learners.
3. identify the strengths, weaknesses, opportunities, and threats of the program.
4. inform future iterations of the curriculum.

## Methods

Using pre- and post-surveys, an outcome-based evaluation [14, 15] was applied to determine if the curriculum was meeting its purpose. The evaluation was guided by the first three levels of Kirkpatrick’s evaluation framework: reaction, learning, and behaviour [16]. Of note, the fourth level, Results, is presently being evaluated through a 3- and 6-month post-evaluation of the full curriculum implementation. Ethical approval for this study was obtained from the Queen’s University and Affiliated Hospitals Health Sciences Research Ethics Board (File #: 6,028,511).

### The educational intervention: the UGME pain management and substance use disorder curriculum

The UGME curriculum was developed by national subject matter experts in education in pain management, opioid prescribing, and opioid use, in collaboration with educational developers, instructional designers, and multimedia specialists [10]. The curriculum consists of six topics identified in Table 1. The curriculum is composed of online modules that have been designed in Articulate 360^®^ software.

**Table 1:** Topics in the UGME curriculum

Topic Number	Title
1	Public Health Perspective
2.1	Core Concepts in Pain
2.2	Core Concepts in Management of Pain
3	Pathophysiology of Pain and Pharmacology of Opioids
4.1	Opioid Prescribing
4.2	Opioid Stewardship in Palliative Care
4.3	Safe Storage and Disposal of Opioids
5.1	Recognizing Opioid Use Disorder
5.2	Managing Opioid Use Disorder
6	Cultural and Legal Considerations for Enhancing Competence

The curriculum is designed to be self-directing or integrated into existing UGME curriculum depending on each of the 17 medical school’s needs and context.

### Setting and participants

Approximately 11,737 UGME students [17] from all 17 Canadian medical schools were invited to participate in the pilot study. Participants were recruited to voluntarily participate through email and social group invitations. All participants received a link to the online consent form and survey via the software program Qualtrics. Participants who completed the pilot received a $50 gift certificate as compensation. The design of the educational curriculum allowed participants to complete the pilot of the 10 online modules at their own pace over a 3-month period. Emails were periodically distributed to participants to inform them of the time remaining to complete the program and served as a reminder to continue working through the program. Post-program surveys were made available to participants once they completed the educational curriculum.

### Data collection

Data for this evaluation were obtained from pre- (*n* = 168) and post-program (*n* = 118) online surveys. The outcome variables that were measured included (a) learning objectives, (b) confidence, (c) value, feasibility, and usability, and (d) strengths, weaknesses, and recommendations.

In both the pre- and post-surveys, participants responded to 72 learning objective statements with the stem: “I am able to…” on a 6-point Likert-type scale, with anchors of 1 (*Strongly disagree*) to 6 (*Strongly agree*). For example, two of the learning objectives in topic 1 are: (i) I am able to describe the epidemiology of pain, and (ii) I am able to describe the health-related and social costs of chronic pain and opioid use in Canada. Participants assessed each of the program’s 72 learning objectives across the 10 modules. Of note, the statements were specific to the learning outcomes identified for each topic. Learners also rated two confidence statements on the same scale (Appendix A).

After completion of the program, participants were asked to reflect on the value, feasibility, and usability of the educational program by completing the post-program survey. Participants responded to six items, using the same 6-point Likert-type scale, that assessed key aspects of the program, including (a) technology, (b) organization, (c) interactivity, (d) visual presentation, (e) ease of use, and (f) presentation of the content (Appendix A). Participants were also given the opportunity to provide feedback through three open-ended questions where they were asked to reflect on the strengths and weaknesses of the program, and to provide recommendations for improving future iterations of the curriculum.

### Data analysis

Descriptive and inferential statistics were used to evaluate participants’ responses to the curriculum. All statistical analyses were conducted using SPSS version 27. Descriptive statistics were used to assess participants’ responses to the value, feasibility, and usability of the program. Differences in the mean scores relating to participants’ confidence and participants’ knowledge of the program learning objectives were assessed using independent t-tests. For each t-test, a Cohen’s d and a percentage change were estimated to quantify the extent of change observed between participants’ pre-and post-training ratings. The analytic samples varied in cases where respondents did not respond to certain questions. Given the large incidence of unmatched pre- and post-intervention scores, differences in mean scores relating to participants’ confidence and participants’ knowledge of the program learning objectives were evaluated using independent t-tests [18]. All missing cases were assumed to be missing at random and were therefore excluded from the analysis. The open-ended survey questions describing the strengths and weakness of the program, and suggested recommendations were qualitatively analysed through open coding and thematic analysis in NVivo 12 [19].

To ensure rigour and trustworthiness of our evaluation process, we engaged in reflexivity [20, 21]. We mitigated biases that could influence the understanding and interpretation of results through regular discussions. This helps ensure that individual and team reflexivity was addressed throughout the research process.

## Results

The results are reported within seven specific components: (i) demographics, (ii) confidence, (iii) knowledge, (iv) value, feasibility, and usability, (v) strengths, (vi) weaknesses, and (vii) recommendations for improving the program.

A total of 168 participants completed the pre-program surveys and 118 (70.24%) post-program surveys.

### Demographics

An overview of the baseline demographic characteristics of the participants (Table 2) indicated that more than one half of the participants identified as a woman (60.1%), were between the ages of 25 and 34 (53.6%) and reported being in their clerkship stage (year 3 or 4) of training (63.1%).

**Table 2:** Participant Demographic Characteristics

Demographic Data	*N*	Percent (%)
*Stage in Medical School*		
Pre-Clerkship	58	34.5
Clerkship	106	63.1
Other	4	2.4
*Age Range*		
18–24	72	42.9
25–34	90	53.6
35–44	6	3.6
*Gender*		
Man	65	38.7
Woman	101	60.1
I do not identify with the gender binary	1	0.6
Prefer not to answer	1	0.6

There was a relatively equal representation of participants across the Canadian medical schools, with a slightly higher number of responses from the Northern Ontario School of Medicine.

### Confidence

Participants’ perceived confidence regarding their knowledge of the use of opioids in the management of pain significantly increased (*t* = -21.13, *p* = 0.000) by 75% between pre-program ($\overline{x}$ = 2.92, *SD* = 1.11) and post-program sessions ($\overline{x}$ = 5.10, *SD* = 0.63). Participants’ perceptions of their confidence to utilize resources to maintain their knowledge of opioids also significantly increased (*t* = -15.85, *p* = 0.000) between pre- ($\overline{x}$ = 3.43, *SD* = 1.15) and post- sessions ($\overline{x}$ = 5.19, *SD* = 0.73) by 51%.

### Knowledge

Overall, participants’ perceived knowledge of the learning objectives significantly increased for all 72 learning objectives across the 10 modules. This paper focuses on those LOs where participants’ perceived knowledge of the learning objective increased by more than 80% (Table 3). Please see Appendix A for a full description of the learning objectives. Findings indicated that participants’ perceived knowledge for seven of the 14 learning objectives for *Topic 6: Cultural and Legal Considerations for Enhancing Competence* improved by over 80%. Similarly, the results showed improvements of over 80% in three out of the six learning objectives for Topic 2.2 (Core Concepts in the Management of Pain), Participants also reported improvements of over 80% for learning objectives in five modules covering topics 3, 4.1, 4.2, 4.3, and 5.2. The findings demonstrated statistically significant increases for all the learning objectives for Topics 1 (Public Health), 2.1 (Core Concepts in Pain), and 5.1 (Recognizing Opioid Use Disorder), but these increases were moderate (less than 80%).

**Table 3:** Learner perceptions of gains in knowledge

Topic	Learning Objective I am able to..	Pre-Program	Post-Program			
		Mean (SD)	Mean (SD)	t	Effect Size (Cohen’s D)	% change
2.2	Describe and interpret the recommendations pertaining to optimization of non-opioid and opioid therapy in the 2017 Canadian Guideline for Opioids for Chronic Non-Cancer Pain.	2.70 (1.18)	5.21 (0.67)	22.681*	1.006	92.96
	Evaluate whether a patient is eligible for opioid therapy to manage their chronic pain.	2.62 (1.08)	5.10 (0.65)	24.243*	0.925	94.66
	Describe how to initiate an opioid trial in a patient with chronic pain and evaluate whether the trial is working.	2.45 (1.13)	5.10 (0.65)	25.114*	0.959	108.16
3	List the four major types of receptor modulation by opioid drugs.	2.55 (1.13)	4.79 (0.98)	17.879*	1.071	87.84
4.1	Describe and apply the entire opioid prescribing cascade, including initiation, titration, switching, and opioid tapering.	2.38 (1.19)	5.23 (0.67)	25.699*	1.013	119.75
4.2	Describe the use of opioids to manage dyspnea and cough.	2.86 (1.21)	5.22 (0.62)	21.612*	1.010	82.52
	Evaluate goals of care and symptom management of people receiving palliative care in the context of the COVID-19 pandemic.	2.85 (1.23)	5.26 (0.62)	21.835*	1.024	84.56
4.3	Name three key messages to help patients understand safe opioid storage and proper disposal practices.	2.75 (1.20)	5.53 (0.57)	26.182*	0.989	101.09
5.2	Describe basic differences between the medications used for OAT.	2.89 (1.25)	5.39 (0.64)	22.128*	1.045	86.51
	Identify and treat opioid withdrawal using appropriate strategies.	2.90 (1.24)	5.25 (0.73)	20.021*	1.061	81.03
6	Discuss the use of opioids in women, particularly those who are pregnant or breast feeding.	2.54 (1.15)	5.28 (0.67)	25.375*	0.983	107.87
	Describe the legal parameters for the prescription of opioids in the jurisdiction of my future practice.	2.43 (1.11)	4.77 (0.92)	19.379*	1.036	96.23
	Review best practices in the management of patients using opioids according to the CMPA.	2.76 (1.20)	4.97 (0.83)	18.446*	1.062	80.07
	Discuss the factors in determining whether a patient on opiates may drive a car.	2.44 (1.06)	5.19 (0.73)	25.953*	0.936	112.71
	Describe three strategies to maintain competence in the treatment of pain within my future practice.	2.54 (1.09)	5.14 (0.68)	24.666*	0.944	102.36
	Discuss how I will monitor my practice to manage patients with issues of substance misuse.	2.62 (1.13)	5.11 (0.70)	22.972*	0.977	95.04
	Outline how to audit my practice of prescribing opioids.	2.26 (1.09)	4.90 (0.85)	23.007*	0.994	116.81

Notes: [1]. *Differences between pre- and post-training scores were tested at *p* < 0.05

[2]. Percentage change calculations were based on pre- and post-training scores

[3]. Cohen’s D uses the pooled standard deviation in calculating effect size

[4]. Percent of improvements are shown for only learning objectives with greater than 80% improvement in perceived knowledge

### Value, feasibility, and usability

Overall, participants reported that the program modules were valuable, feasible, and usable. Over 90% of participants ‘*agreed’* or ‘*strongly agreed*’ that the modules were well-organized (*M* = 5.41, *SD* = 0.70), interactive (*M* = 5.42, *SD* = 0.67), visually pleasing (*M* = 5.42, *SD* = 0.72), easy to use (*M* = 5.37, *SD* = 0.78), and presented at a level that was easy for participants to understand (*M* = 5.48, *SD* = 0.68). However, only 75% of participants, ‘*agreed*’ or ‘*strongly agreed*’ that the technology used to access the modules worked well (*M* = 5.02, *SD* = 1.00).

### Strengths of the program

The overall strengths of the program included clear and comprehensive content, an interactive and organized design, and relevance to future clinical practice.

#### Clear and comprehensive content

Participants discussed how the comprehensiveness of the program content addressed key gaps in existing educational offerings for UGME learners. More specifically, they expressed appreciation for the clear, evidence-informed, and up-to-date information contained within the modules. Participants noted the comprehensive nature of the program in relation to both the breadth of topics covered and the detailed information provided on each topic. As one participant commented: *“[The curriculum] covers pretty well everything you could possibly think of regarding opioids (from history to mechanisms to prescribing to storage, etc.).”* Some participants also found value in the resources that were provided throughout the program. For example, one participant highlighted: *“[There is] tremendous detail in each module. Everything was thoroughly explained, and a number of resources are offered throughout for users to access if they are seeking additional information.”* Through the comprehensiveness, participants perceived that the program enhanced their knowledge of important topics regarding the diagnosis, treatment, and management of opioid use disorder.

#### Interactive and organized design

Most participants found the curriculum interactive and well-organized. Participants appreciated the logical organization of the modules and visually aesthetic nature of the content. As one participant commented: *“The information is presented very clearly, [and] in a logical order from module to module so that it is possible to better understand the knowledge acquired in one module in relation to that acquired in previous modules.”*

Several participants also emphasized that the interactivity of the learning platform enhanced learner engagement with the program content. Participants expressed appreciation for the use of multi-media approaches (e.g., audio, video, readings) and the provision of links to external resources. Participants discussed how the inclusion of such resources encouraged further learning and development, as evident by the comment: *“I appreciated the link to external sources so that we can continue to be up to date.”*

Another strength of the program design was the use of various formats of questions such as true/false, multiple choice, and short answer questions, that were embedded within the modules. Participants described how these questions provided valuable opportunities to consolidate their knowledge and actively engage with the program content. As one participant noted: *“There was a lot of interaction, which is much more engaging than having the information presented to us without any involvement on our part.”* Overall, participants expressed how the design of the curriculum positively contributed to the quality of their learning experience.

#### Relevant for future clinical practice

Participants also highlighted the relevance and applicability of the content. Several participants noted that the scenarios and case-studies were insightful and applicable future clinical work. This was captured in the following extract:

*I liked how the last few modules placed an emphasis on case-based scenarios. I thought that this allowed us to think through the knowledge that we had been given and then explain how we would use this newfound knowledge to manage/treat/assess a patient.*

Further, participants stated that the inclusion of a diversity of persons with lived experiences greatly contributed to the quality of the curriculum. Indeed, participants emphasized the importance of including content specifically focused on Indigenous communities, elderly populations, and pregnancy.

### Weaknesses of the program

The overall weakness of the program included the length, repetition of content, the lack of clarity and relevance of the assessment questions, technology and French translation issues.

#### Length and repetition

A common concern challenge expressed by the participants related to the length and time requirements to complete the full curriculum. Some participants noted that the time requirements for the course were challenging to accommodate in their schedule. While many participants recognized how the comprehensive content was valuable, some cautioned that the length may hinder the quality of the learning experience as expressed by one participant:

*The biggest weakness of the Opioid program is the length of the program. While I appreciate the thoroughness of the program, the amount of information makes it difficult to focus and understand what the most important aspects of the program are.*

#### Lack of clarity and relevance of assessment questions

Participants raised concerns with the number, type, and quality of questions within the modules. Some participants felt that the quiz questions were not always representative of the material within the module, while others felt that there were not enough questions. Additionally, some participants expressed concerns with the clarity and length of the open-ended questions. As one participant noted:

*I would also be in favour of changing short answer questions to [a] series of MCQs [Multiple Choice Questions]. Students tend to give a more thoughtful answer to an MCQ whereas they may just skip over a long answer question.*

#### Technological issues

Some participants reported challenges with accessing and navigating the online platform, including indicators of program progress and activity completion, missing portable document formats (PDFs), as well as issues with voice overs, and loading and playing a few of the videos. Some participants also reported issues with the compatibility of modules with different devices and browsers. Participants emphasized that technological concerns impacted the quality of the learning experience. For example, one participant commented: *“After completing [the] post-module tests, it doesn’t redirect the user back to the module sets or to the next module… It was a bit frustrating to use when this happened…”*.

### Recommendations

Participants offered suggestions for improving the program that included streamlining content, addressing technology issues, and enhancing the clarity and relevance of assessment questions embedded in each of the modules.

#### Streamline content

One of the most frequently cited recommendations was to streamline the program content to reduce the length of both individual modules and the overall program. Participants suggested that it would be worthwhile for program developers to examine the content for potential overlap and repetition. As one participant noted: *“The length of the program needs to be reduced. The content is super valuable and well presented, there just simply is too much of it for this one program…”* Participants highlighted that streamlining the program content may help to enhance the quality of the learning experience and may improve the integration of the program content into existing educational programs.

#### Address technological concerns

Participants suggested that addressing the technological issues would be beneficial. Specifically, participants recommended that it would be worthwhile to (a) ensure that the PDFs in each module are downloadable, (b) improve the quality of images, audio-clips and videos, and (c) enhance the ease of navigation with the software platform.

#### Enhance the clarity and relevance of assessment questions

Participants offered several suggestions for improving the quality of the assessment questions within modules. These included, (a) ensuring that the were no identical pre- and post-test questions, (b) revising open-ended questions to enhance their clarity, (c) providing more clinically relevant questions (as compared to questions that only require recall), and (d) including additional questions throughout the modules. Participants indicated that these changes to the assessment questions would enhance learners’ engagement with the material and provide greater opportunities for learners to consolidate and apply their knowledge. One participant elaborated on the need for different pre- and post-test questions:

*Make the pre-test questions different from the post-test. It is the nature of medical students (and students in general) as we are so very busy, that when we realize the post-test (an indicator of our learning) questions are the same as that of the pre-test, we tend to skim through the content of the module and just find the answers that will allow us to do better on the post-test, without focusing too much on learning the content in the module that we know is not being tested.*

#### Include diverse experiences and perspectives

Lastly, participants discussed the potential benefits of including a diverse range of population-specific and context-specific lived experiences of patients and health care providers. Participants noted that those diverse experiences and perspectives would better contextualize the program content. Moreover, participants suggested that the inclusion of additional diverse practical examples (e.g., cases, scenarios) would help learners to consolidate their knowledge and apply their knowledge in real-world contexts. Participants expressed that this would be beneficial for their current education and for their future clinical practice. For instance, one participant commented: *“…the modules could possibly be enhanced by adding testimonials from people related to the material presented.”*

## Discussion

The economic, social, and health burdens associated with the opioid epidemic are high, which make it imperative that we identify ways to address this public health crisis. The findings from the program evaluation of the pilot for the AFMC Pain Management and Substance Use Disorder curriculum suggest that this program meets a need in current UGME curricula. A 2009 Canada-wide study conducted by Watt-Watson and colleagues found that the majority of health sciences faculties and departments could not identify the number of hours dedicated to pain management within their curriculum, and that there was a very broad range of responses from across schools [22]. Almost two decades later, Tran et al. found that most students could only recall receiving seven hours of formal education on pain management in either pre-clerkship or clerkship, and they reported that it had been primarily delivered in didactic formats [23].

Introducing new content into UGME curriculums is a difficult balancing act as instruction time is often already stretched to accommodate existing requirements. Participants’ concerns regarding the amount of time spent to complete the modules is likely related to the fact that they were asked to complete all six topics (10 modules) for the pilot program. We anticipate that as medical schools across Canada implement this program, they will be able to adapt it to the needs of their institution. Schools that already have more pain management content in their curricula can choose which modules to integrate into existing courses and how to integrate them in a thoughtful and meaningful way. These could be completed asynchronously by learners, or more likely as part of a blended learning model. Blended learning has been found to be comparable to traditional learning formats in terms of learner outcomes, and in some cases has been shown to be more effective for learners [24–26].

Student feedback from the module indicates that the comprehensive, standardized content was a key strength of the program for learners. Learners appreciated the clear, evidence-informed, and up-to-date information in the modules which they thought addressed gaps in their current curricula. Learners also indicated that curricular elements including use of case-studies [27, 28] which covered diverse lived experiences helped them to see how the content would be applicable to their future practice. Case studies are viewed as important learning strategies and with the recent technological advances (e.g., ChatGPT) they can further support individualizing the case studies to specific learner needs and continue to promote self-regulated and self-directed learning opportunities [29]. The design features of the module, including the organization of content, and the interactive design elements and links to additional resources were also viewed as curricular strengths. The curriculum was designed to address and meet the Accessibility for Ontarians with Disabilities Act standards [30] to ensure the module features were easily assessable and organized.

Participants wanted more assessment questions embedded in the modules to help test their learning. This suggests they are using the assessment questions as formative learning opportunities to guide them in what they know well and what they need to review. Technology challenges were also cited as a weakness—some a result of the learning management platform and some resulting from end-user. Given this, it is important to have technology support available to address any registered user difficulties in accessing the program and/or it’s learning features. As a result of this evaluation, changes were made with respect to the content, formative learning activities, and user support prior to the full implementation of this pain management and substance use disorder curriculum.

### Limitations

This national program was designed to be delivered in Canada’s two official languages, French and English. We received a much smaller number of Francophone participants than Anglophone participants. Concerns with the quality of the translation identified early in the pilot study may have caused attrition with some Francophone participants. As well, we had representative students from all 17 Canadian medical schools who participated in the pilot study; however, these participants were volunteers and therefore may not be representative of the average learner within each of these programs.

### Next steps

A one-year post-implementation evaluation is being planned to identify how each school has integrated the modules within their curricula. AFMC has also just implemented a postgraduate medical education curriculum and a continuing professional development program for practicing physicians that scaffolds this learning to other educational contexts. These are presently being evaluated.

## Conclusion

Learners who completed the new AFMC National UGME Pain Management and Substance Use Disorder Curriculum demonstrated increased knowledge and confidence in their ability to discuss the opioid epidemic, provide treatment for pain management, prescribe, store and dispose of opioids, discuss the pathophysiology of pain and the pharmacology of opioids, and identify, manage and treat opioid use disorders. The new curriculum fills the gaps in existing pain management curricula and contributes to the efforts at addressing the current opioid pandemic in Canada.

### Electronic supplementary material

Below is the link to the electronic supplementary material.

Supplementary Material 1

## Data Availability

The datasets used and/or analysed during the current study are available from the corresponding author on reasonable request.

## Abbreviations

AFMC: Association of Faculties of Medicine of Canada
PDFs: Portable Document Formats
UGME: Undergraduate Medical Education
