# Supplementary material for: Developing a national undergraduate medical education pain management and substance use disorder curriculum to address the opioid crisis: a program evaluation pilot study
Source: BMC Med Educ. 2024 Mar 8;24:258. doi: 10.1186/s12909-024-05181-z (PMC10921711; doi:10.1186/s12909-024-05181-z)
Supplement: Supplementary file 1 — Supplementary Material 1 [file 12909_2024_5181_MOESM1_ESM.docx]

**Appendix A: Post Program Survey**

**Section 1: Knowledge and Content**

1. Were your educational needs addressed by the program?

- Yes
- No

2. Why were your educational needs **not** met?

3. Please indicate your level of agreement with each of the following statements:

3a. Topic 1: The Public Health Perspective

Please indicate your level of agreement with the following statements:

|  | Strongly Disagree | Disagree | Slightly Disagree | Slightly Agree | Agree | Strongly Agree |
| --- | --- | --- | --- | --- | --- | --- |
| 1. I am confident in my knowledge of opioids. |  |  |  |  |  |  |
| 1. I am confident in utilizing resources to maintain my knowledge on opioids. |  |  |  |  |  |  |

|  | Strongly Disagree | Disagree | Slightly Disagree | Slightly Agree | Agree | Strongly Agree |
| --- | --- | --- | --- | --- | --- | --- |
| 1. I am able to describe the epidemiology of pain. |  |  |  |  |  |  |
| 1. I am able to describe the epidemiology opioid prescribing for pain. |  |  |  |  |  |  |
| 1. I am able to describe the health-related and social costs of chronic pain and opioid use in Canada. |  |  |  |  |  |  |
| 1. I am able to describe the epidemiology of the “opioid epidemic” including the overlap between pain and opioid misuse/addiction. |  |  |  |  |  |  |
| 1. I am aware of the historical context for the opioid crisis. |  |  |  |  |  |  |
| 1. I am able to understand the impacts of pain, opioid misuse, and opioid use disorder on psychosocial functioning. |  |  |  |  |  |  |
| 1. I am able to identify interventions at the community level. |  |  |  |  |  |  |
| 1. I am able to describe the role of medical students and physicians in advocating for community interventions. |  |  |  |  |  |  |

3b. Topic 2.1: Core Concepts in Pain
Please indicate your level of agreement with the following statements:

|  | Strongly Disagree | Disagree | Slightly Disagree | Slightly Agree | Agree | Strongly Agree |
| --- | --- | --- | --- | --- | --- | --- |
| 1. I am able to define acute pain and describe its epidemiology. |  |  |  |  |  |  |
| 1. I am able to define chronic pain and understand the concept of chronic pain as a disease in itself rather than simply a symptom. |  |  |  |  |  |  |
| 1. I am able to identify the global burden of chronic pain and its epidemiology. |  |  |  |  |  |  |
| 1. I am able to understand the neurobiology of chronic pain as a complex biopsychosocial and spiritual experience. |  |  |  |  |  |  |
| 1. I am able to describe the link between acute and chronic pain and how transient pain progresses into persistent pain. |  |  |  |  |  |  |
| 1. I am able to identify the risk factors for chronic pain and interventions to reduce the risk of developing chronic pain. |  |  |  |  |  |  |
| 1. I am able to Identify biological and psychosocial processes involved in the development of chronic pain. |  |  |  |  |  |  |

3c. Topic 2.2: Core Concepts in Management of Pain  
Please indicate your level of agreement with the following statements:

|  | Strongly Disagree | Disagree | Slightly Disagree | | Slightly Agree | Agree | Strongly Agree |
| --- | --- | --- | --- | --- | --- | --- | --- |
| 1. I am able to describe and interpret the recommendations pertaining to optimization of non-opioid and opioid therapy in the 2017 Canadian Guideline for Opioids for Chronic Non-Cancer Pain. |  |  |  |  | |  |  |
| 1. I am able to articulate the most appropriate treatment plan for a patient in pain using psychological and nonpharmacological approaches, including preventive interventions. |  |  |  |  | |  |  |
| 1. I am able to determine the most appropriate treatment plan for a patient in pain using pharmacological non-opioid and procedural approaches, including preventive interventions. |  |  |  |  | |  |  |
| 1. I am able to effectively communicate with patients and their family/supports to facilitate understanding of the risks (e.g. Opioid use disorder, among other adverse effects) and the possible benefits of prescription medications, including opioids. |  |  |  |  | |  |  |
| 1. I am able to evaluate whether a patient is eligible for opioid therapy to manage their chronic pain. |  |  |  |  | |  |  |
| 1. I am able to describe how to initiate an opioid trial in a patient with chronic pain and evaluate whether the trial is working. |  |  |  |  | |  |  |

3d.  Topic 3: Pathophysiology of Pain and Pharmacology of Opioids

Please indicate your level of agreement with the following statements:

|  | Strongly Disagree | Disagree | Slightly Disagree | Slightly Agree | Agree | Strongly Agree |
| --- | --- | --- | --- | --- | --- | --- |
| 1. I am able to explain the basic mechanisms of pain signals. |  |  |  |  |  |  |
| 1. I am able to explain how the descending pathway modulates pain signals. |  |  |  |  |  |  |
| 1. I am able to define different types of pain. |  |  |  |  |  |  |
| 1. I am able to explain the absorption, distribution, and metabolism of opioid drugs. |  |  |  |  |  |  |
| 1. I am able to provide examples of short- and long-lasting opioids. |  |  |  |  |  |  |
| 1. I am able to explain and provide examples of dosing equivalencies. |  |  |  |  |  |  |
| 1. I am able to explain how the actions of opioids are mediated through opioid receptors. |  |  |  |  |  |  |
| 1. I am able to explain the differences between the different types of opioid receptors. |  |  |  |  |  |  |
| 1. I am able to list the four major types of receptor modulation by opioid drugs. |  |  |  |  |  |  |
| 1. I am able to explain the role of opioid receptors in mediating side effects. |  |  |  |  |  |  |
| 1. I am able to explain the concepts of tolerance, dependence, and withdrawal. |  |  |  |  |  |  |

3e. Topic 4.1: Opioid Stewardship
Please indicate your level of agreement with the following statements:

|  | Strongly Disagree | Disagree | Slightly Disagree | Slightly Agree | Agree | Strongly Agree |
| --- | --- | --- | --- | --- | --- | --- |
| 1. I am able to recognize the immediate relevance of social and historical context for opioid prescribing. |  |  |  |  |  |  |
| 1. I am able to apply the concept of morphine equivalence to identify dose related risks of opioids. |  |  |  |  |  |  |
| 1. I am able to describe and apply the entire opioid prescribing cascade, including initiation, titration, switching, and opioid tapering. |  |  |  |  |  |  |

3f.  Topic 4.2: Opioid Stewardship in Palliative Care
Please indicate your level of agreement with the following statements:

|  | Strongly Disagree | Disagree | Slightly Disagree | Slightly Agree | Agree | Strongly Agree |
| --- | --- | --- | --- | --- | --- | --- |
| 1. I am able to describe the use of opioids to manage dyspnea and cough. |  |  |  |  |  |  |
| 1. I am able to describe the relationship between goals of care and symptom management to improve quality of life. |  |  |  |  |  |  |
| 1. I am able to explain the concept of “total pain” and the need for an interdisciplinary treatment plan using non-pharmacological and pharmacologic approaches. |  |  |  |  |  |  |
| 1. I am able to identify symptom and functional assessment tools that are used in palliative and end of life care. |  |  |  |  |  |  |
| 1. I am able to identify routes of opioid administration other than oral formulation to administer for symptom management at the end of life. |  |  |  |  |  |  |
| 1. I am able to describe the effects of liver and renal dysfunction on opioid metabolism and excretion. |  |  |  |  |  |  |
| 1. I am able to identify the importance of, and role played by, caregivers in palliative and end of life care. |  |  |  |  |  |  |
| 1. I am able to evaluate goals of care and symptom management of people receiving palliative care in the context of the COVID-19 pandemic. |  |  |  |  |  |  |

3g. Topic 4.3: Safe Storage and Disposal of Opioids
Please indicate your level of agreement with the following statements:

|  | Strongly Disagree | Disagree | Slightly Disagree | Slightly Agree | Agree | Strongly Agree |
| --- | --- | --- | --- | --- | --- | --- |
| 1. I am able to describe the risks associated with unsafe opioid storage and disposal practices. |  |  |  |  |  |  |
| 1. I am able to identify best practices for safe medication storage and disposal. |  |  |  |  |  |  |
| 1. I am able to name three key messages to help patients understand safe opioid storage and proper disposal practices. |  |  |  |  |  |  |

3h. Topic 5.1: Recognizing Opioid Use Disorder
Please indicate your level of agreement with the following statements:

|  | Strongly Disagree | Disagree | Slightly Disagree | Slightly Agree | Agree | Strongly Agree |
| --- | --- | --- | --- | --- | --- | --- |
| 1. I am able to identify risk factors for opioid use disorder. |  |  |  |  |  |  |
| 1. I am able to differentiate between tolerance, withdrawal, physiologic dependence, and opioid use disorder |  |  |  |  |  |  |
| 1. I am able to explain the consequences of opioid misuse on the health of individuals, and related public health concerns. |  |  |  |  |  |  |
| 1. I am able to understand the utility of various assessment tools to assess the risk of misuse in patients being considered for opioid therapy. |  |  |  |  |  |  |
| 1. I am able to recognize warning signs in a patient who is using opioids. |  |  |  |  |  |  |
| 1. I am able to utilize a trauma-informed approach when communicating with patients with opioid use disorder. |  |  |  |  |  |  |
| 1. I am able to recognize barriers to care originating from bias and stigma for people with substance use disorders and the importance of non-stigmatizing language. |  |  |  |  |  |  |

3i. Topic 5.2: Managing Opioid Use Disorder
Please indicate your level of agreement with the following statements:

|  | Strongly Disagree | Disagree | Slightly Disagree | Slightly Agree | Agree | Strongly Agree |
| --- | --- | --- | --- | --- | --- | --- |
| 1. I am able to identify treatments and follow-up strategies for managing patients with inappropriate opioid use, including rehabilitation and psychosocial approaches focused on wellness, behavior modification and harm reduction. |  |  |  |  |  |  |
| 1. I am able to identify the principles of opioid agonist therapy (OAT). |  |  |  |  |  |  |
| 1. I am able to describe basic differences between the medications used for OAT. |  |  |  |  |  |  |
| 1. I am able to identify challenges in communication (e.g., diagnosis of substance use disorder, disagreement with patient around dosing) and apply strategies to overcome them. |  |  |  |  |  |  |
| 1. I am able to identify and treat opioid withdrawal using appropriate strategies. |  |  |  |  |  |  |

3j. Topic 6: Cultural and Legal Considerations for Enhancing Competence
Please indicate your level of agreement with the following statements:

|  | Strongly Disagree | Disagree | Slightly Disagree | Slightly Agree | Agree | Strongly Agree |
| --- | --- | --- | --- | --- | --- | --- |
| 1. I am able to describe the unique challenges of prescribing opioids in the elderly population. |  |  |  |  |  |  |
| 1. I am able to discuss the use of opioids in women, particularly those who are pregnant or breast feeding. |  |  |  |  |  |  |
| 1. I am able to identify the challenges in prescribing pain medications for patients with hepatic and/or renal insufficiency. |  |  |  |  |  |  |
| 1. I am able to recognize the bias that may be evident in the treatment of Indigenous Peoples with opioid use disorder and discuss anti-bias strategies. |  |  |  |  |  |  |
| 1. I am able to identify the social determinants that contribute to the issues of substance misuse in First Nations, Inuit and Metis populations |  |  |  |  |  |  |
| 1. I am able to describe the legal parameters for the prescription of opioids in the jurisdiction of my future practice. |  |  |  |  |  |  |
| 1. I am able to review best practices in the management of patients using opioids according to the CMPA. |  |  |  |  |  |  |
| 1. I am able to discuss the factors in determining whether a patient on opiates may drive a car. |  |  |  |  |  |  |
| 1. I am able to describe three strategies to maintain competence in the treatment of pain within my future practice. |  |  |  |  |  |  |
| 1. I am able to discuss how I will monitor my practice to manage patients with issues of substance misuse. |  |  |  |  |  |  |
| 1. I am able to outline how to audit my practice of prescribing opioids. |  |  |  |  |  |  |
| 1. I am able to discuss the role that the industry and others have had in influencing practices of prescribing opioids. |  |  |  |  |  |  |
| 1. I am able to describe the risks and consideration for prescribing opioids to post-operative patients. |  |  |  |  |  |  |
| 1. I am able to describe the importance of providing culturally safe care to Indigenous Peoples and how I can implement culturally safe care into my practice |  |  |  |  |  |  |

**Section 3: Value, Feasibility, and Utility of Modules**

4. Please indicate your level of agreement with the following statements:

|  | Strongly Disagree | Disagree | Slightly Disagree | Slightly Agree | Agree | Strongly Agree |
| --- | --- | --- | --- | --- | --- | --- |
| 1. The technology used to access the modules worked well. |  |  |  |  |  |  |
| 1. The modules were well organized. |  |  |  |  |  |  |
| 1. The modules were interactive. |  |  |  |  |  |  |
| 1. The content of the modules was visually pleasing. |  |  |  |  |  |  |
| 1. The modules were easy to use. |  |  |  |  |  |  |
| 1. The content of the modules was presented at a level I could understand |  |  |  |  |  |  |

Q5. What are the strengths of this Opioid program?

Q6. What are the weaknesses of this Opioid program?

Q7. What recommendations do you have for improving this program?

**Section 3: Demographics**
Q8. What year of medical school are you currently in?

- Pre-clerkship
- Clerkship
- Other, please specify

Q9. How do you self-identify in terms of gender?

- Man
- Woman
- I do not identify with gender binary
- Prefer not to answer

Q10. What is your age range?

- <18
- 18-24
- 25-34
- 35-44
- 45-54
- 55-65
- >65

Q11. Which School of Medicine do you attend?

- University of Alberta Faculty of Medicine and Dentistry
- Cumming School of Medicine
- University of British Columbia Faculty of Medicine
- University of Manitoba College of Medicine
- Memorial University of Newfoundland Faculty of Medicine
- Dalhousie University Faculty of Medicine
- McMaster University Michael G. DeGroote School of Medicine
- Northern Ontario School of Medicine
- Queen's University School of Medicine
- Western University Schulich School of Medicine & Dentistry
- University of Ottawa Faculty of Medicine
- University of Toronto Faculty of Medicine
- Université Laval Faculté de Médecine
- McGill University Faculty of Medicine
- Université de Montréal Faculté de Médecine
- Université de Sherbrooke Faculté de Médecine et des sciences de la santé
- University of Saskatchewan College of Medicine
- Other, please specify

**Appendix B: Post Module Survey Example**

**AFMC Topic 1: The Public Health Perspective**

**Section 1: Knowledge and Content**

1. As a result of this module, please indicate your level of agreement with the following statements:

|  | Strongly Disagree | Disagree | Slightly Disagree | Slightly Agree | Agree | Strongly Agree |
| --- | --- | --- | --- | --- | --- | --- |
| 1. I am able to describe the epidemiology of pain. |  |  |  |  |  |  |
| 1. I am able to describe the epidemiology of pain. |  |  |  |  |  |  |
| 1. I am able to describe the health-related and social costs of chronic pain and opioid use in Canada. |  |  |  |  |  |  |
| 1. I am able to describe the epidemiology of the “opioid epidemic” including the overlap between pain and opioid misuse/addiction. |  |  |  |  |  |  |
| 1. I am aware of the historical context for the opioid crisis. |  |  |  |  |  |  |
| 1. I am able to understand the impacts of pain, opioid misuse, and opioid use disorder on psychosocial functioning. |  |  |  |  |  |  |
| 1. I am able to identify interventions at the community level. |  |  |  |  |  |  |
| 1. I am able to describe the role of medical students and physicians in advocating for community interventions. |  |  |  |  |  |  |

|  |  |
| --- | --- |

Q2. Please indicate your level of agreement with the following statements:

|  | Strongly Disagree | Disagree | Slightly Disagree | Slightly Agree | Agree | Strongly Agree |
| --- | --- | --- | --- | --- | --- | --- |
| 1. The module met my expectations. |  |  |  |  |  |  |
| 1. The length of the module was appropriate. |  |  |  |  |  |  |
| 1. The objectives were clearly defined. |  |  |  |  |  |  |
| 1. The content will be useful in my work environment. |  |  |  |  |  |  |
| 1. The module has increased my comfort in understanding the role of Public Health in Opioid Response. |  |  |  |  |  |  |
| 1. The module increased my knowledge about Public Health with regards to Opioid Response. |  |  |  |  |  |  |
| 1. I will apply what I have learned in this module to my work environment. |  |  |  |  |  |  |
| 1. This module was valuable to my professional growth. |  |  |  |  |  |  |
| 1. I am satisfied with this module. |  |  |  |  |  |  |
| 1. This module was useful for my current medical program. |  |  |  |  |  |  |
| 1. This module was useful for preparing for the Medical Council of Canada (MCC) exam. |  |  |  |  |  |  |
| 1. I will recommend this module to others. |  |  |  |  |  |  |
| 1. Through this module, I gained new knowledge. Please specify. |  |  |  |  |  |  |
| 1. Through this module, I gained new skills. Please specify. |  |  |  |  |  |  |

**Section 2: Value, Feasibility, and Utility of Module**

Q3. Please indicate your level of agreement with the following statements:

|  | Strongly Disagree | Disagree | Slightly Disagree | Slightly Agree | Agree | Strongly Agree |
| --- | --- | --- | --- | --- | --- | --- |
| 1. The technology used to access the module worked well. |  |  |  |  |  |  |
| 1. The module was well organized. |  |  |  |  |  |  |
| 1. The module was interactive. |  |  |  |  |  |  |
| 1. The module content was visually pleasing. |  |  |  |  |  |  |
| 1. The module was easy to use. |  |  |  |  |  |  |
| 1. The content was presented at a level I could understand. |  |  |  |  |  |  |

Q4. What are the strengths of this module?

Q5. What are the weaknesses of this module?

Q6. What suggestions do you have for improving this module?

Q7. Please enter the number of approximate hours it took you to complete this module:

**Section 3: Demographics**
 Q8. What year of medical school are you currently in?

- Pre-clerkship
- Clerkship
- Other, please specify

Q9. What is your age range?

- <18
- 18-24
- 25-34
- 35-44
- 45-54
- 55-65
- >65

Q10. How do you self-identify in terms of gender?

- Man
- Woman
- I do not identify with gender binary
- Prefer not to answer

Q11. Which School of Medicine do you attend?

- University of Alberta Faculty of Medicine and Dentistry
- Cumming School of Medicine
- University of British Columbia Faculty of Medicine
- University of Manitoba College of Medicine
- Memorial University of Newfoundland Faculty of Medicine
- Dalhousie University Faculty of Medicine
- McMaster University Michael G. DeGroote School of Medicine
- Northern Ontario School of Medicine
- Queen's University School of Medicine
- Western University Schulich School of Medicine & Dentistry
- University of Ottawa Faculty of Medicine
- University of Toronto Faculty of Medicine
- Université Laval Faculté de Médecine
- McGill University Faculty of Medicine
- Université de Montréal Faculté de Médecine
- Université de Sherbrooke Faculté de Médecine et des sciences de la santé
- University of Saskatchewan College of Medicine
- Other, please specify
